# Improving bioaccessibility and physicochemical property of blue-grained wholemeal flour by steam explosion

**DOI:** 10.3389/fnut.2022.877704

**Published:** 2022-07-27

**Authors:** Feng Kong, Qinghua Zeng, Yue Li, Yang Zhao, Xingfeng Guo

**Affiliations:** College of Agronomy, Liaocheng University, Liaocheng, China

**Keywords:** steam explosion, wholemeal, bioaccessibility, digestibility, rheological property

## Abstract

Whole grain contains many health-promoting ingredients, but due to its poor bioaccessibility and processibility, it is not widely accepted by consumers. The steam explosion was exploited to modify the nutritional bioaccessibility and the physicochemical properties of wholemeal flour in this study. *In vitro* starch digestibility, *in vitro* protein digestibility of wholemeal flour, total flavonoids content, and total phenolics content of digestive juice were used to evaluate the bioaccessibility, and a significant variation (*p* < 0.05) was noted. Results showed that steam explosion enhanced the gastric protein digestibility ranged from 5.67 to 6.92% and the intestinal protein digestibility ranged from 16.77 to 49.12%. Steam-exploded wholemeal flour (0.5 MPa, 5 min) had the highest protein digestibility and rapidly digestible starch content. Compared with native flour, steam explosion (0.5 MPa, 5 min) contributed to a 0.72-fold and 0.33-fold increment of total flavonoids content and total phenolics content in digestible juice. Chemical changes of wholemeal flour, induced by steam explosion, caused the changes in the solvent retention capacity, rheological property of wholemeal flour, and altered the falling number (and liquefaction number). An increasing tendency to solid-like behavior and the gel strength of wholemeal flour was significantly enhanced by the steam explosion at 0.5 MPa for 5 min, while the gluten was not weakened. This study indicated that steam-exploded wholemeal flour (0.5 MPa, 5 min) could serve as a potential ingredient with the noticeable bioaccessibility and physicochemical properties in cereal products.

## Introduction

Wheat is one of the most important grain crops worldwide, which provides the essential dietary components for many individuals daily, such as energy, proteins, dietary fibers, and phytochemicals (1). The consumption of wholemeal has been associated with the prevention of chronic diseases, including cardiovascular diseases and diabetes (2). Despite the health-promoting ingredients associated with wholemeal, there were nutritional and technological challenges in consuming it as a staple food (3). The technological unfavorable effects and poor bioaccessibility of wholemeal resulted mostly from the insoluble part of bran (4–6). Wheat bran contained aleurone as a monolayer of cells overlying the endosperm, which was rich in dietary fiber and phenolic compounds (7). However, dietary fiber mainly included unextractable arabinoxylans found in plant cell walls, and the bioactive compounds were encapsulated from intact cells (7–9). The lower nutritional bioaccessibility was associated with intact cells (10, 11), the cell walls reduced the permeability of enzymes and the release of nutrients due to the encapsulation of nutrients inside the food structure (12–15). These characteristics resulted in the poor bioaccessibility of wholemeal. And due to the insoluble part of bran exited, wholemeal in cereal-based food processing with poor processibilities, such as reduced dough elasticity (16). Therefore, increasing the soluble part of arabinoxylans and breaking the intact cell wall might be the efficient strategies to improve the bioaccessibility and physicochemical properties of wholemeal.

Thermal treatments could increase the soluble dietary fiber content and the bioaccessibility of bioactive compounds from cereal bran together with some technological benefits (17, 18). Recently, researchers have shown an increased interest in steam explosion applications. The steam explosion was a novel hydrothermal processing technology in the food industry with high efficiency and low energy consumption, which was usually employed in high-fiber materials (5, 19, 20). The steam explosion was employed to modify the physicochemical structure of the feedstock, resulting in a partial rupture of cell walls, with the consequent release of intracellular components and change in the functional property of final products (5, 6, 21, 22). Therefore, steam explosion treatment may be a potential strategy to improve the bioaccessibility and processibility of wholemeal flour in the food industry. However, the effects of steam explosion on the biophysicochemical properties of wholemeal remain to be demonstrated.

This study aimed to investigate the effect of steam explosion on the bioaccessibility of wholemeal flour, which was evaluated by starch digestibility, protein digestibility of wholemeal flour, total flavonoids content, and total phenolics content of digestive juice. And the effects of steam explosion on the physicochemical properties of wholemeal flour, including color profiles, chemical compounds, solvent retention capacity, and rheological properties were analyzed.

## Materials and methods

### Sample preparation

**FIGURE 1:**
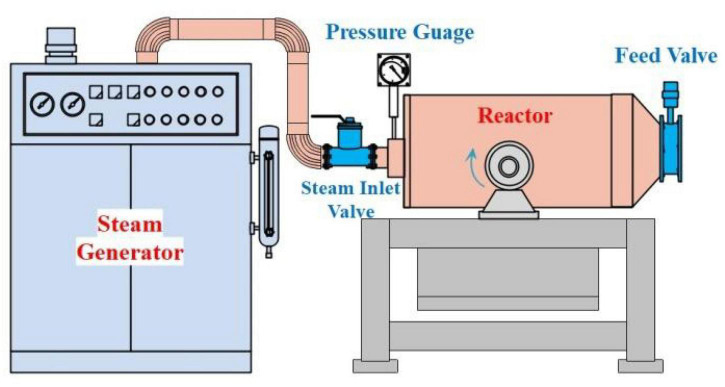
Schematic diagram of the experimental apparatus for the steam explosion.

The steam explosion was performed using a self-designed batch vessel (Figure 1), which consisted of a reaction chamber (WY19, Big Soldier Food Machinery, Henan, China) and a steam generator (WY19, Big Soldier Food Machinery, Henan, China). Blue-grained wheat kernel (harvested from the test site of Liaocheng University, Liaocheng, China) was loaded into the reactor chamber and treated at 0.3–0.7 MPa for 3–7 min. The reaction system was then terminated with a sudden explosion by opening the feed valve, and the wheat was allowed to dry at 60°C for 12 h. The dried wheat sample (100 g) was ground for 2 min in a ZT-150 high-speed grinder (Yongkang Zhanfan Industry and Trade Co., Ltd., Zhejiang, China).

### *In vitro* protein digestion of wholemeal flour

Method (23, 24) adopted with modifications was considered to evaluate the *in vitro* protein digestibility following gastric (pepsin) and intestinal (trypsin) digestion. Briefly, 2 g of wholemeal flour was mixed with 20 ml of distilled water, and added 1 mol/L HCl to adjust pH = 2, then incubated with 1 g of pepsin (P6322, >3,000 U/mg, Shanghai Macklin Biochemical Co., Ltd., Shanghai, China) in 100 ml of 0.01 mol/L HCl at 37°C for 2 h and neutralized with 1 mol/L NaHCO_3_. This was followed by the addition of 0.4 g of trypsin (pig pancreas, 250 USP U/mg, Yuanye Biotechnology Co., Ltd., Shanghai, China), 2.5 g of bile powder in 100 ml of 0.1 mol/L NaHCO_3_, and incubated at 37°C for 2 h.

### *In vitro* starch digestion of wholemeal flour

*In vitro* starch digestibility was determined by the previous report (25–29) with modifications. α-Amylase (6 g, 35 U/mg, Shanghai Macklin Biochemical Co., Ltd., Shanghai, China) and 40 ml of deionized water were mixed and followed by stirring for 10 min. Then the mixed solution was centrifuged at 4,000 rpm for 15 min. Finally, 30 ml of the supernatant was mixed with 2 ml of amyloglucosidase (from *Aspergillus niger*, 1 × 10^5^ U/ml, Shanghai Macklin Biochemical Co., Ltd., Shanghai, China) and 3 ml of deionized water to prepare digestive enzyme solution. Wholemeal flours (500 mg) were mixed with 14 ml of deionized water and boiled in a water bath for 5 min. Then 1 ml of digestive enzyme solution was added, shaken, and incubated at 37°C. Sample solutions (0.2 ml) were added with 80% of ethanol solution (5 ml) immediately, after 20, 60, 120, 180, and 240 min, respectively.

### Antioxidant compounds and activity of digestive juice

The total phenolics content of digestive juice after protein digestion was analyzed using the Folin-Ciocalteu colorimetric method described by Veronica et al. (30). The total flavonoids content of digestive juice after protein digestion was analyzed by the method (31). The DPPH radical scavenging activity of digestive juice after protein digestion was analyzed by the previous method (5).

### Chemical compounds of wholemeal flour

The protein content of wholemeal flour was analyzed using the AACC method 46-08. Total arabinoxylans and water-extractable arabinoxylans content were determined using the Hashimoto’s method (32). The starch content of wholemeal flour was measured by the Goñi’s method (25) with modifications. Briefly, wholemeal flours (0.1 g) were mixed with 2 mol/L of KOH solution (6 ml) and shaken at 25°C for 30 min. Then, 98% of acetum (1 ml), 0.4 mol/L of sodium acetate buffer solution (pH = 4.75, 3 ml), and amyloglucosidase (from *Aspergillus niger*, 1 × 10^5^ U/ml, 200 μl) were added and incubated at 60°C for 45 min.

### The Hagberg-Perten falling number analysis of wholemeal flour

The Hagberg-Perten falling number (FN) of all samples was determined using an FN-IV instrument (Hangzhou Daji Electric Instrument Co., Ltd., Hangzhou, China) according to the manufacturer’s instructions. Liquefaction number (LN) was calculated using the following formula: LN = 6,000/(FN-50).

### Color measurement of wholemeal flour

The color profiles of native and steam-exploded wholemeal flours were measured with a chromameter (Minolta CR-10, Japan). L* means lightness of the flour, a* indicates green or red-purple color, and b* indicates yellow or blue color (33). Chroma (34) and hue (35) were calculated using the L*, a*, and b* values.

### Solvent retention capacity of wholemeal flour

The solvent retention capacity (SRC) tests that included 5% lactic acid (LA), 5% sodium carbonate (SC), 50% sucrose (Suc), and water (W) were measured according to the AACC International Approved Method 56-11.02.

### Rheological property analysis of wholemeal flour

The dynamic rheological property of all samples was investigated using an HR-1 Rheometer (TA Instruments, United States) based on a method (6, 36). An amount of 4 g of flours and 25 ml of deionized water were mixed and shaken at 95°C water for 30 min and stored at 4°C. After pasting and cooling, portions of each sample were transferred directly onto the parallel plate (40 mm in diameter, Peltier plate steel). The sweep procedure was run at 0.1–1,000 rad/s at 25°C with a strain amplitude of 2%.

### Statistical analysis

Three replicated tests were performed, and the average values were reported. Experimental data were processed by one-way analysis of variance using IBM SPSS Statistics 20 (IBM, NY, United States) with Duncan’s multiple range test (*p* < 0.05). The results were reported as mean ± standard deviation.

## Results and discussion

### Protein bioaccessibility of wholemeal flour

**FIGURE 2:**
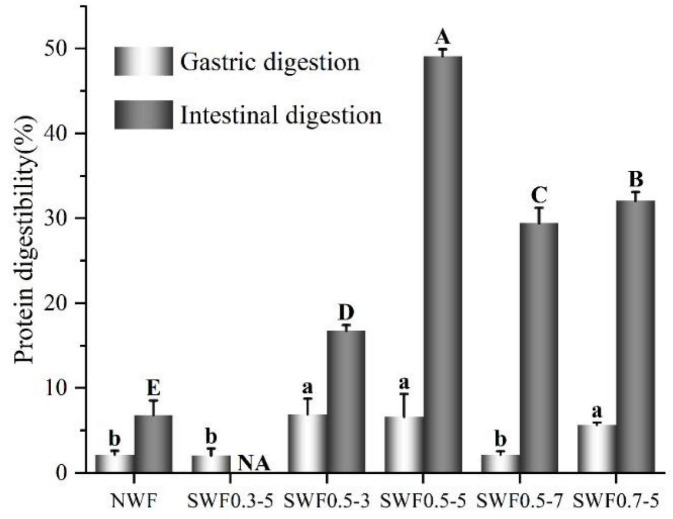
Effect of steam explosion on *in vitro* protein digestibility of wholemeal flour. NWF, native wheat flour; SWF, steam-exploded wheat flour; SWF0.3-5, SWF at 0.3 MPa for 5 min; SWF0.5-3, SWF at 0.5 MPa for 3 min; SWF0.5-5, SWF at 0.5 MPa for 5 min; SWF0.5-7, SWF at 0.5 MPa for 7 min; SWF0.7-5, SWF at 0.7 MPa for 5 min; NA, not available. (Means that do not share a letter are significantly different at *p* < 0.05.).

The effect of steam explosion on bioaccessibility can be evaluated *in vitro* by the simulation of gastric and intestinal digestion, which estimated the bioaccessibility changes resulting from the variations in the steam explosion processing (7, 37). Protein bioaccessibility at the gastric stage and intestinal stage during the *in vitro* gastrointestinal digestion of wholemeal flours is shown in Figure 2. During the gastric stage, protein digestibility of native wheat flour was 2.16%, while steam-exploded flours exhibited protein digestibility ranging between 2.05 and 6.92%, the highest protein digestibility was noted for samples subjected to steam explosion treatment at 0.5 MPa for 3 min (6.92%), 0.5 MPa for 5 min (6.63%), and 0.7 MPa for 5 min (5.67%), and differences between the steam explosion at 0.5 MPa for 3 min and 5 min were minimal. However, steam-exploded wheat flour at 0.3 MPa for 5 min and 0.5 MPa for 7 min showed no statistical variation compared with that of native wholemeal flour (*p* > 0.05). During the intestinal stage, protein digestibility of wheat flour ranged from 6.81 to 49.12%. Protein digestibility of native wheat flour (6.81%) increased significantly (*p* < 0.05) after the steam explosion. The highest ultimate protein digestibility (49.12%) of steam-exploded wholemeal flour was obtained under the condition of the steam explosion at 0.5 MPa for 5 min. The steam explosion broke cell walls and promoted the breakdown of proteins into smaller units, which might improve the digestibility and bioaccessibility of wheat flour (5, 21, 38, 39). In this study, the results of *in vitro* protein digestibility showed that steam explosion was an effective strategy that could be employed to improve protein bioaccessibility in wholemeal flour.

### Starch bioaccessibility of wholemeal flour

**FIGURE 3:**
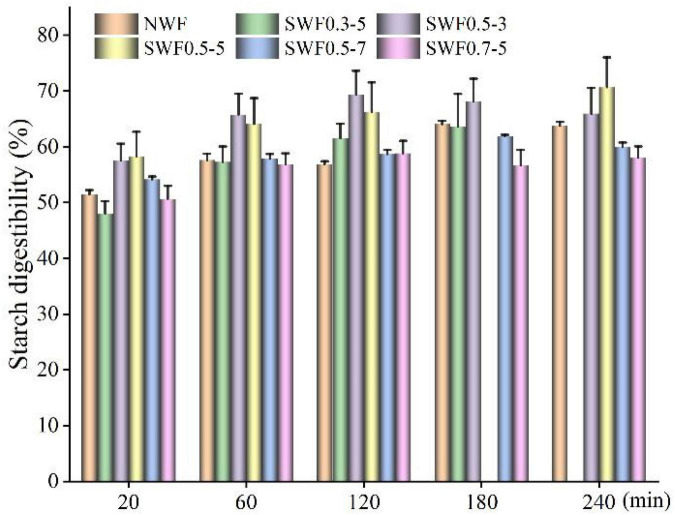
Effect of steam explosion on *in vitro* starch digestibility of wholemeal flour. NWF, native wheat flour; SWF, steam-exploded wheat flour; SWF0.3-5, SWF at 0.3 MPa for 5 min; SWF0.5-3, SWF at 0.5 MPa for 3 min; SWF0.5-5, SWF at 0.5 MPa for 5 min; SWF0.5-7, SWF at 0.5 MPa for 7 min; SWF0.7-5: SWF at 0.7 MPa for 5 min.

Starch was one of the main nutrients in wheat, and its digestibility directly influenced the digestion and absorption of flour (40). In the *in vitro* digestion process (Figure 3), the enzymatic hydrolysis rate of all wholemeal flours increased rapidly in the first 20 min and stabilized after 60 min, which was in line with human digestible characteristics (41). Wholemeal flour formed a more unstable structure after being saturated with steam with high-pressure and high-temperature treatment. The highest hydrolysis rate of wholemeal starch was between 0 and 20 min. In this period, the hydrolysis rate of starch in native wholemeal was 2.57% starch/min, and the highest hydrolysis rate of starches was found in steam-exploded wholemeal flour at 0.5 MPa for 5 min (2.92% starch/min), followed by 0.5 MPa for 3 min (2.88% starch/min), 0.5 MPa for 7 min (2.71% starch/min), 0.3 MPa for 5 min, and 0.7 MPa for 5 min (2.40–2.53% starch/min). From 20 to 60 min, there was a remarkable drop in the hydrolysis rate, particularly for steam-exploded wholemeal at 0.5 MPa for 7 min (0.09% starch/min), whereas native flour was 0.15% starch/min.

The rapidly digestible starch, slowly digestible starch, and resistant starch contents of native wholemeal flour were 51.47, 5.43, and 43.10%, respectively (Table 1). The rapidly digestible starch content of wholemeal increased significantly to 57.60–58.31% (*p* < 0.05) by the steam explosion at 0.5 MPa for 3–5 min, whereas slowly digestible starch and resistant starch content decreased to 7.92–11.77% and 30.63–33.77%, respectively. A proper and reasonable steam explosion disrupted the cell wall structure and promoted enzyme access starch, and the starch was gelatinized during steam explosion processing, thus enhancing starch susceptibility and bioaccessibility. Wholemeal modification by the steam explosion at 0.5 MPa for 5 min had the highest rapidly digestible starch (58.31%), lowest slowly digestible starch (7.92%), and resistant starch (33.77%) contents, respectively.

**TABLE 1:** Rapidly digestible starch, slowly digestible starch, and resistant starch content of native and steam-exploded wholemeal flour.

Samples	Rapidly digestible starch (%)	Slowly digestible starch (%)	Resistant starch (%)
NWF	51.47 ± 0.94*^bc^*	5.43 ± 0.63*^d^*	43.10 ± 0.61*^a^*
SWF0.3-5	48.05 ± 2.69*^c^*	13.50 ± 0.56*^a^*	38.45 ± 3.18*^ab^*
SWF0.5-3	57.60 ± 3.63*^a^*	11.77 ± 1.61*^b^*	30.63 ± 5.22*^c^*
SWF0.5-5	58.31 ± 5.36*^a^*	7.92 ± 1.20*^c^*	33.77 ± 6.51*^bc^*
SWF0.5-7	54.17 ± 0.59*^ab^*	4.52 ± 0.39*^d^*	41.31 ± 0.92*^a^*
SWF0.7-5	50.68 ± 2.80*^bc^*	8.14 ± 0.64*^c^*	41.17 ± 2.74*^a^*

NWF, native wheat flour; SWF, steam-exploded wheat flour; SWF0.3-5, SWF at 0.3 MPa for 5 min; SWF0.5-3, SWF at 0.5 MPa for 3 min; SWF0.5-5, SWF at 0.5 MPa for 5 min; SWF0.5-7, SWF at 0.5 MPa for 7 min; SWF0.7-5, SWF at 0.7 MPa for 5 min. Means that do not share a letter are significantly different at *p* < 0.05.

### Bioaccessibility of antioxidant compounds in wholemeal flour

**FIGURE 4:**
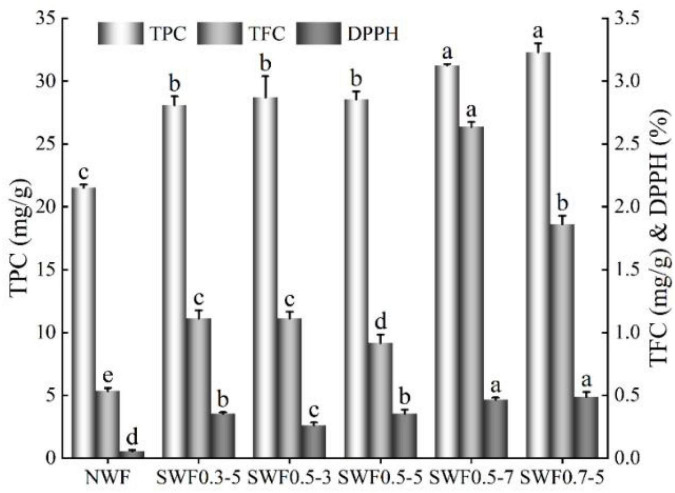
Effect of steam explosion on antioxidant compounds and activity of digestive juice. NWF, native wheat flour; SWF, steam-exploded wheat flour; SWF0.3-5, SWF at 0.3 MPa for 5 min; SWF0.5-3, SWF at 0.5 MPa for 3 min; SWF0.5-5, SWF at 0.5 MPa for 5 min; SWF0.5-7, SWF at 0.5 MPa for 7 min; SWF0.7-5, SWF at 0.7 MPa for 5 min; TPC, total phenolics content; TFC, total flavonoids content; DPPH, DPPH radical scavenging activity. Means that do not share a letter are significantly different at *p* < 0.05.

Phenolics could increase the reactive antioxidant potential and subsequently decrease the risk of free radical-related diseases (42). The healthy effect of phenolic acids was dependent on their bioaccessibility and bioavailability (7). The thermal treatment was conducive to release the bioactive compounds from food, especially from high-fiber foods, and improve their antioxidant capacity *in vitro* (43). Steam explosion significantly (*p* < 0.05) enhanced total phenolics, flavonoids contents, and DPPH radical scavenging activity (Figure 4). Phenolics and flavonoids bioaccessibility of digestible juice after steam explosion treatment were 1.30–1.50 and 1.72–4.94 times of those native wholemeal digestible juice. The results indicated that steam explosion improved the antioxidant compounds’ bioaccessibility. Steam explosion significantly increased the free and conjugated ferulic acid content in the wheat bran (44) and enhanced the release of bound phenolic compounds of barley bran (45) and soybean seed coat phenolic profiles (46). The steam explosion could form a large cavity and intercellular space, which aided the release of phenolic profiles and enhanced the antioxidant activities (5, 21, 46, 47). The bioavailability of these compounds strongly depended on their bioaccessibility, which could be affected by the processing (2). Bioactive compounds were encapsulated from intact cells and that steam explosion could have a significant effect of the high-shear and high temperature on cell integrity and bioaccessibility, which might influence the fraction that was made available for intestinal absorption (7).

### Chemical compositions of wholemeal flour

No significant differences (*p* > 0.05) in protein and starch content were found among the native and all steam-exploded wholemeal flours (Table 2). The protein and starch contents of wholemeal flours ranged from 14.88 to 16.77% and 59.42 to 66.67%, respectively. Arabinoxylans and water-extractable arabinoxylans content of different samples were shown in Table 2, which of native wholemeal were 5.04 and 0.48%, respectively, while that of steam-exploded wholemeal were 4.91–5.95 and 0.53–0.74%, respectively. The water-extractable arabinoxylans content was used to evaluate the effect of the treatment on cell wall degradation (48). It demonstrated that the water-extractable arabinoxylans content was significantly (*p* < 0.05) increased to reach a peak (0.74%) by steam explosion. The water-extractable arabinoxylans content increased with increasing pressure to reach a peak at 0.7 MPa for 5 min. At 0.5 MPa, the content showed a peak at 0.5 MPa for 5 min, and after 5 min, it decreased. The outer layer of the wheat kernel was surrounded by the thick non-lignified cell walls, which were composed mainly of dietary fiber, with the predominance of arabinoxylans (7). The lower starch digestibility/bioaccessibility was associated with intact cells of cereal (11), which encapsulated intracellular starch and delayed digestive enzyme access. The steam explosion could promote the conversion of arabinoxylans to water-extractable arabinoxylans, it broke the crystalline structure of the dietary fiber, which validated that the steam explosion could hydrolyze hemicelluloses (49). As a result, the steam explosion might improve the starch digestibility/bioaccessibility of wholemeal. The water-extractable arabinoxylans with the functional properties of higher water absorption and higher viscosity, and the suitable content of arabinoxylans was beneficial for the dough extensibility and subsequent product quality (50–52).

**TABLE 2:** Proximate composition of native and steam-exploded wholemeal flour.

Samples	AX (%)	WEAX (%)	Protein (%)	Starch (%)	FN	LN
NWF	5.04 ± 0.04*^c^*	0.48 ± 0.00*^f^*	14.88 ± 1.56*^a^*	60.60 ± 0.88*^ab^*	342.33 ± 16.25*^a^*	20.57 ± 1.13*^b^*
SWF0.3-5	5.25 ± 0.05*^b^*	0.53 ± 0.01*^e^*	16.06 ± 0.32*^a^*	66.67 ± 1.43*^a^*	292.67 ± 31.39*^ab^*	24.99 ± 3.06*^b^*
SWF0.5-3	5.12 ± 0.12*^bc^*	0.54 ± 0.01*^d^*	15.29 ± 0.93*^a^*	59.42 ± 4.21*^ab^*	274.33 ± 24.21*^bc^*	26.94 ± 2.75*^b^*
SWF0.5-5	5.24 ± 0.05*^b^*	0.66 ± 0.00*^b^*	16.77 ± 1.18*^a^*	60.26 ± 5.41*^ab^*	231.50 ± 60.10*^c^*	30.14 ± 11.71*^b^*
SWF0.5-7	4.91 ± 0.09*^d^*	0.60 ± 0.00*^c^*	15.95 ± 0.98*^a^*	63.98 ± 1.06*^ab^*	74.50 ± 6.36*^d^*	253.45 ± 65.83*^a^*
SWF0.7-5	5.95 ± 0.03*^a^*	0.74 ± 0.00*^a^*	15.85 ± 1.56*^a^*	61.28 ± 2.79*^ab^*	73.67 ± 4.51*^d^*	259.76 ± 51.06*^a^*

NWF, native wheat flour; SWF, steam-exploded wheat flour; SWF0.3-5, SWF at 0.3 MPa for 5 min; SWF0.5-3, SWF at 0.5 MPa for 3 min; SWF0.5-5, SWF at 0.5 MPa for 5 min; SWF0.5-7, SWF at 0.5 MPa for 7 min; SWF0.7-5, SWF at 0.7 MPa for 5 min; AX, arabinoxylans; WEAX, water-extractable arabinoxylans; FN, falling number; LN, liquefaction number. Different letters indicated significant differences at *p* < 0.05 in the same column.

The falling number apparatus has been widely used in the rapid determination of α-amylase activity in grain, which was quantified by reducing the viscosity of the flour paste (53). The falling number value of wholemeal flour was determined by the different conditions of steam explosion (Table 2). The result indicated that the flour paste liquefied quickly when the intensity of the steam explosion was increased. Compared with native wholemeal flour, the falling number of wholemeal flours significantly (*p* < 0.05) decreased by the steam explosion to reach a peak at 0.7 MPa for 5 min. Liquefaction numbers could be used in the milling and baking industries to calculate the mixing ratio of flour to be blended with a known falling number (54, 55). Liquefaction number was a linear evaluation of α-amylase activity in grain, and there were no significant changes (*p* > 0.05) in liquefaction number found among the native and all steam-exploded wholemeal flours at 0.3 MPa for 5 min and 0.5 MPa for 3–5 min. In general, the data suggested that the steam explosion might enhance the susceptibility of starch to digestive enzyme hydrolysis and increase the damage of wheat starch, which together contributed to the decrease of the falling number values.

### Color measurement of wholemeal flour

**FIGURE 5:**
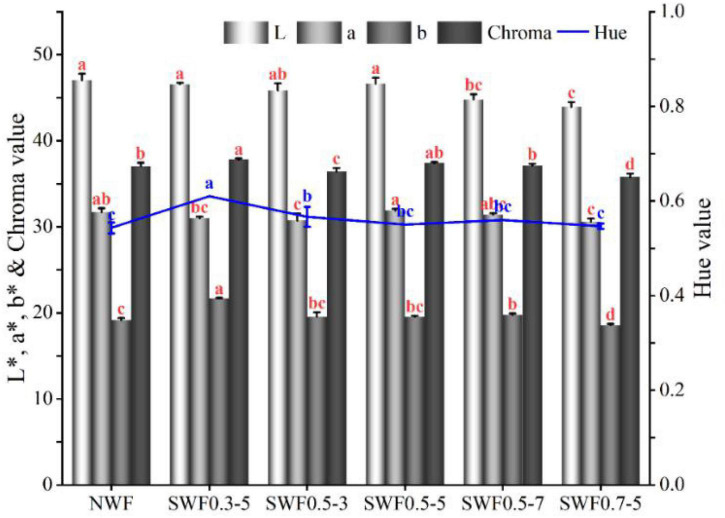
Effect of steam explosion on color profile of blue-grained wheat flour. NWF, native wheat flour; SWF, steam-exploded wheat flour; SWF0.3-5, SWF at 0.3 MPa for 5 min; SWF0.5-3, SWF at 0.5 MPa for 3 min; SWF0.5-5, SWF at 0.5 MPa for 5 min; SWF0.5-7, SWF at 0.5 MPa for 7 min; SWF0.7-5, SWF at 0.7 MPa for 5 min. Means that do not share a letter are significantly different at *p* < 0.05.

The color profiles of L*, a*, and b* values of wholemeal flours are shown in Figure 5. L*, a*, and b*values denoted lightness to darkness, redness to greenness, and yellowness to blueness, respectively (56). The steam-exploded wholemeal samples exhibited no change in L* values without the sample at 0.5 MPa for 7 min and 0.7 MPa for 5 min and showed no change in a* values without the sample at 0.5 MPa for 3 min and 0.7 MPa for 5 min and b* values without the sample (0.3 MPa for 5 min, 0.5 MPa for 7 min, and 0.7 MPa for 5 min), compared to native wholemeal flour (L* = 47.03, a* = 31.70, b* = 19.17). Color measurements indicated that the severe conditions of steam explosion treatment on wholemeal at 0.5 MPa for 7 min and 0.7 MPa for 5 min yielded a darker color (lower L* values were 44.80 and 43.93). The undesired Maillard browning reaction, caramelization, and oxidation product formation could be responsible for the decrease of lightness in steam-exploded flours compared to native powder (6, 22). The a* values of the steam-exploded wholemeal ranged from 30.60 to 31.90, and the b* values ranged from 18.57 to 21.67. The chroma values indicated the purity or saturation and showed no significant variation compared to native powder (34). No change was found in chroma between native and steam-exploded wholemeal flours at 0.5 MPa for 5–7 min (*p* > 0.05), which indicated the stability of color in wholemeal flours. The Hue angle values increased from 0.54 to 0.57–0.61 during the steam explosion process at 0.3 MPa for 5 min and 0.5 MPa for 3 min. It suggested increment from a greener color to an orange-red color of steam-exploded wholemeal. These results indicated that the partial steam explosion affected the color quality of wholemeal flour and produced more browning compound(s). There was a remarkable positive relationship (*p* < 0.01) between b* and Hue value (*r* = 0.948). The steam explosion at 0.5 MPa for 5 min had better color performance than other conditions, which was conducive in maintaining the color acceptability of native wholemeal.

### Solvent retention capacity of wholemeal flour

**FIGURE 6:**
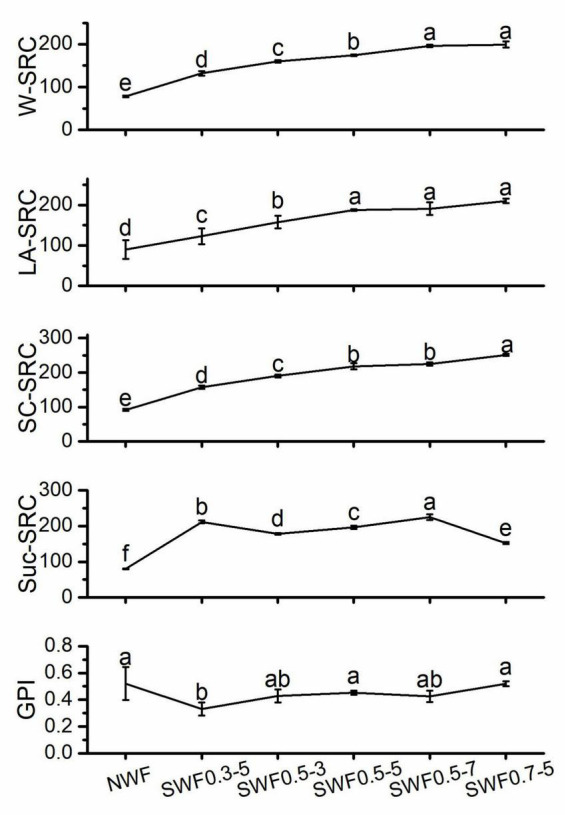
Effect of steam explosion on solvent retention capacity of wheat flour. NWF, native wheat flour; SWF, steam-exploded wheat flour; SWF0.3-5, SWF at 0.3 MPa for 5 min; SWF0.5-3, SWF at 0.5 MPa for 3 min; SWF0.5-5, SWF at 0.5 MPa for 5 min; SWF0.5-7, SWF at 0.5 MPa for 7 min; SWF0.7-5, SWF at 0.7 MPa for 5 min; GPI, gluten performance index. Means that do not share a letter are significantly different at *p* < 0.05.

Solvent retention capacity (SRC) was deemed to be an efficient method to evaluate wheat flour quality; it is a special method for predicting the flour function by estimating the relative contributions of individual flour compounds to water absorption, which is also used to evaluate oat flour properties (57). The differences in the SRC values between native and steam-exploded wholemeal flour were observed, and the SRC values were significantly (*p* < 0.05) increased by the steam explosion (Figure 6). W-SRC significantly (*p* < 0.05) increased with the steam explosion treatment time extended at 0.5 MPa from 3 to 7 min. W-SRC also increased with the extension of steam explosion treatment pressure from 0.3 to 0.7 MPa for 5 min. This might be caused by the fracture of wholemeal under the high pressure and high temperature of steam explosion, which exposed more hydrophilic groups and allowed the flour more water-binding capacity (6). Steam explosion enhanced the Suc-SRC value compared with that of the native, which might be owing to the water-extractable arabinoxylans content increased by steam explosion. Suc-SRC significantly (*p* < 0.05) increased with the steam explosion treatment time extended at 0.5 MPa from 3 to 7 min. However, Suc-SRC decreased with the increase in pressure from 0.3 to 0.7 MPa for 5 min. Compared to native flour, LA-SRC was significantly (*p* < 0.05) increased by steam explosion. The LA-SRC of steam-exploded flour showed no change when the treatment was at 0.5 MPa for 5 min. SC-SRC increased significantly (*p* < 0.05) with the extending of steam explosion pressure from 0.3 to 0.7 MPa for 5 min, which might be attributed to the increase of damaged starch content induced by steam explosion. GPI might be a proper predictive index of the gluten property influenced by all the components in wheat flour (58). The steam explosion did not change significantly (*p* > 0.05) on the GPI of wholemeal flour, except for the condition at 0.3 MPa for 5 min, which showed that steam explosion was conducive to maintaining the gluten strength. According to the results above, the steam explosion was beneficial in improving the SRC values of wholemeal flour while the gluten was not weakened.

### Rheological property of wholemeal flour

**FIGURE 7:**
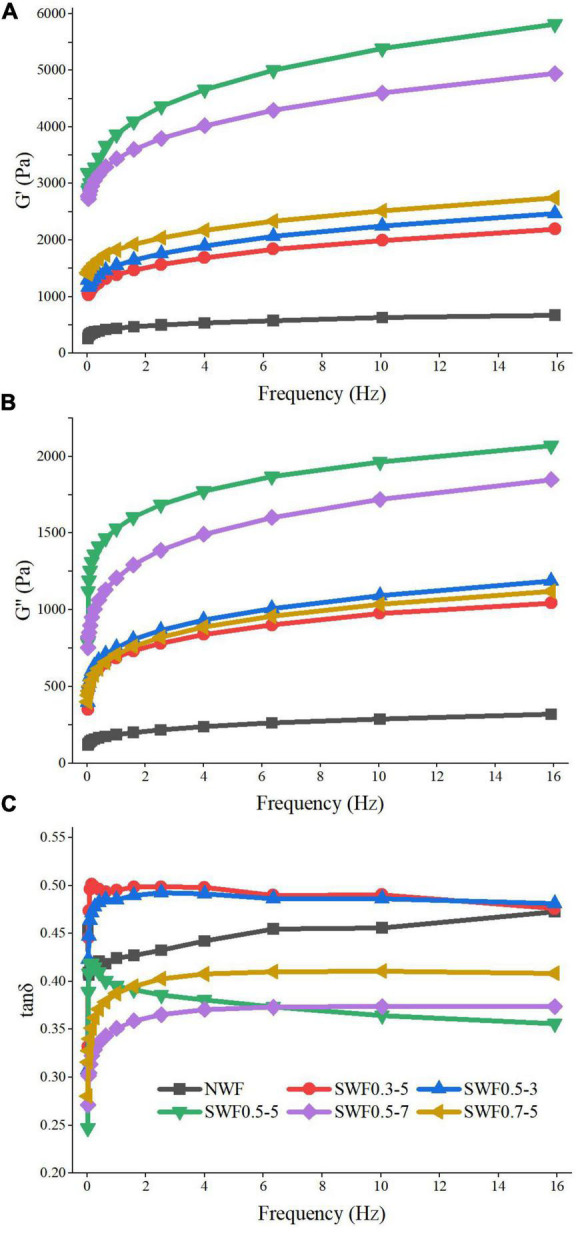
Effect of the steam explosion on the rheological property of wholemeal flour. **(A)** G’ of samples; **(B)** G” of samples; **(C)** tan δ of samples; NWF, native wheat flour; SWF, steam-exploded wheat flour; SWF0.3-5, SWF at 0.3 MPa for 5 min; SWF0.5-3, SWF at 0.5 MPa for 3 min; SWF0.5-5, SWF at 0.5 MPa for 5 min; SWF0.5-7, SWF at 0.5 MPa for 7 min; SWF0.7-5, SWF at 0.7 MPa for 5 min.

Both the storage modulus and the loss modulus of wholemeal flour increased with the increase of frequency, and the elasticity and viscosity were frequency-dependent (59). The storage modulus and loss modulus were increased by the steam explosion compared with native, which might be attributed to the porous structure and consequently a solvent retention capacity in steam-exploded wholemeal flour. The values of both the storage modulus and loss modulus of native wheat flour increased through the frequency range, which indicated that wholemeal flour behaved more like soft gels (60). Rheological evaluation (Figures 7A,B) revealed an increasing tendency to solid-like behavior after steam explosion treatment, and the steam explosion increased the gel strength of wholemeal flour. Steam explosion treatment was conducive in modifying the natural viscoelastic properties of wholemeal flour gel, and the highest storage modulus and loss modulus of steam-exploded flour gel were at 0.5 MPa for 5 min. The tanδ is the ratio of the loss modulus to the storage modulus; the larger the tanδ value, the higher the viscosity and smaller the elasticity of the gel (61). The steam explosion remained the inherent rheological property, with the same trend of storage modulus being greater than loss modulus (6). The tanδ value of all wholemeal samples was less than 1, steam explosion at 0.5 MPa for 5–7 min and 0.7 MPa for 5 min decreased the tanδ value of wholemeal flour, and the tanδ value of steam-exploded wholemeal flour at 0.5 MPa for 5 min showed the lowest value (Figure 7C). In this case, it indicated that the steam-exploded wholemeal flour gel was more elastic than the native flour. The results showed that the solid property of wholemeal flour was increased by the steam explosion at 0.5 MPa for 5 min.

### Correlation of various parameters of wholemeal flour

**FIGURE 8:**
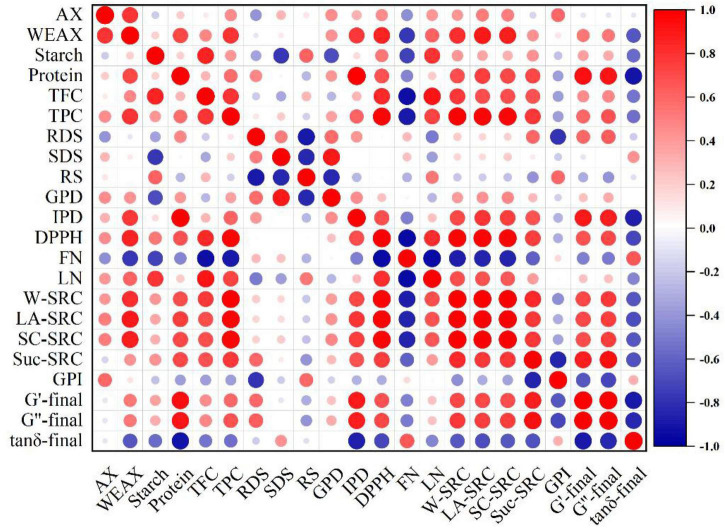
Pearson’s correlation between bioaccessibility and flour characteristics of wholemeal flour. AX, arabinoxylans; WEAX, water-extractable arabinoxylans; FN, falling number; LN, liquefaction number; TPC, total phenolics content; TFC, total flavonoids content; DPPH, radical scavenging activity; RDS, rapidly digestible starch; SDS, slowly digestible starch; RS, resistant starch; GPI, gluten performance index; GPD, gastric protein digestibility; IPD, intestinal protein digestibility.

Elucidating the correlations of bioaccessibility and physicochemical properties of wholemeal flour as shown in Figure 8 might be conducive in developing an indirect processing strategy to control the flour characteristics according to the changes in conditions in the steam explosion. Total phenolics exhibited significant (*p* < 0.05) correlations with the DPPH radical scavenging activity of wholemeal extracts. A positive correlation was found between the Suc-SRC and loss modulus (*r* = 0.93, *p* < 0.05). The water-extractable arabinoxylans and total phenolics content were significantly (*p* < 0.05) correlated with LA-SRC. Total phenolics content was positively related to the W-SRC (*r* = 0.99), LA-SRC (*r* = 0.97), and SC-SRC (*r* = 0.98), whereas negatively connected to the falling number (*r* = –0.89). Protein content was positively related to the intestinal protein digestion, viscosity, storage modulus, and loss modulus, whereas negatively connected to the loss factor. The falling number showed a strong negative relationship with the liquefaction number, while the falling number positively correlated with the total flavonoids content.

## Conclusion

The bioaccessibility and physicochemical properties of wholemeal flour were altered by the thermal-mechanical action of steam explosion. The cell wall was broken by facilitating the conversion of water-unextractable to water-extractable arabinoxylans. As a result, steam explosion treatment improved protein digestibility, starch digestibility, and phenolics bioaccessibility of wholemeal flour. Compared with native wholemeal flour, steam-exploded wholemeal flour existed the highest protein digestibility and the hydrolysis rate of starches at 0.5 MPa for 5 min. And steam explosion contributed to the increase of total flavonoids content, total phenolics content, and DPPH radical scavenging activity in digestible juice. Above chemical changes in wholemeal flour induced by steam explosion caused the physicochemical property changes of wholemeal flour, including the color profiles, solvent retention capacity, and rheological property. Results showed steam explosion was beneficial in improving the solvent retention capacity values while the gluten was not weakened. An increasing tendency to solid-like behavior and the gel strength of wholemeal flour was significantly enhanced by the steam explosion at 0.5 MPa for 5 min. These phenomena demonstrated that steam explosion induced physicochemical property changes of wholemeal flour depending on the chemical constituent change, such as arabinoxylans and starch. This study provided an effective strategy that could be employed to improve the nutritional bioaccessibility and modify the physicochemical property of cereal flour.

## Data availability statement

The original contributions presented in the study are included in the article/supplementary material, further inquiries can be directed to the corresponding authors.

## Author contributions

FK and XG: conceptualization, resources, and supervision. FK, YL, and QZ: methodology and validation. FK: software, formal analysis, data curation, writing-original draft preparation, writing-review and editing, project administration, and funding acquisition. QZ, FK, YZ, and YL: investigation. QZ and FK: visualization. All authors have read and agreed to the published version of the manuscript.
